# Mandibular Metastasis From Colon Cancer Presenting as Jaw Pain

**DOI:** 10.7759/cureus.93377

**Published:** 2025-09-27

**Authors:** Osama Faridi, Hajra Jamil, Faisal Mehmood, Jose Machain

**Affiliations:** 1 Medicine, Midwestern University Arizona College of Osteopathic Medicine, Glendale, USA; 2 Internal Medicine, Services Institute of Medical Sciences, Lahore, PAK; 3 Gastroenterology, HonorHealth Deer Valley Medical Center, Phoenix, USA; 4 Gastroenterology, HonorHealth Osborn Medical Center, Scottsdale, USA

**Keywords:** adenocarcinoma, colonoscopy, jaw pain, liver lesions, metastasis, sigmoid mass

## Abstract

Colorectal cancer (CRC) may rarely present with atypical symptoms, particularly in the context of metastatic disease. While the most common sites of metastasis include the liver, lungs, peritoneum, and lymph nodes, osseous metastases are uncommon, and mandibular involvement is exceedingly rare. We report a case of a 57-year-old male who presented with several weeks of isolated jaw pain. Imaging revealed a destructive mass in the right mandible, and histopathology confirmed metastatic adenocarcinoma of colorectal origin. Abdominal imaging and colonoscopy identified a fungating, partially obstructing sigmoid mass with synchronous liver metastases. Due to extensive disease, surgical resection was not pursued. The patient was initiated on systemic chemotherapy with folinic acid, 5-fluorouracil, and oxaliplatin (FOLFOX) and bevacizumab, with early biochemical and clinical response. This case demonstrates that oral metastasis of CRC portends a poor prognosis. Therefore, high clinical suspicion, timely diagnosis, and initiation of systemic therapy are essential to optimize patient outcomes and maintain quality of life.

## Introduction

Colorectal cancer is the third most diagnosed cancer worldwide, with over 1.9 million cases annually [1]. Early diagnosis is critical, yet up to 20% of patients present with metastatic disease at the time of diagnosis [2]. Metastasis commonly occurs in the lymph nodes, liver, lungs, and peritoneum [3]. Rarely, metastatic CRC manifests symptoms related to distant, non-visceral sites, such as the bones, making timely diagnosis particularly challenging.

Oral metastatic tumors account for approximately 1% of all malignant neoplasms of the jaw [4]. The mandible, though anatomically distant, can occasionally serve as the initial site of metastatic disease and may be mistaken for benign odontogenic or inflammatory conditions. The majority of metastatic lesions are detected in patients in their fifth to seventh decades. Approximately 6.6% of metastatic jawbone lesions have been reported to originate from the colon or rectum. The mandible is the most frequently involved location of metastasis in the jaw bones, with the molar area the most frequently involved site [5,6]. We present a case with an atypical presentation of metastatic CRC, where jaw pain served as the first manifestation of metastatic adenocarcinoma of the colon. This case was presented by the corresponding author of this article as a poster at the American College of Gastroenterology annual meeting in Philadelphia, Pennsylvania, in October 2024 [7].

## Case presentation

A 57-year-old male presented to the hospital with several weeks of persistent jaw pain. His medical history was otherwise unremarkable. He reported longstanding alterations in bowel habits over the past few years but had not sought prior medical evaluation. He denied experiencing abdominal pain, melena, hematochezia, unintentional weight loss, or fatigue. He denied any recent dental infections or having undergone any dental procedures before presentation. There was no known family history of colorectal cancer, and a screening colonoscopy performed seven years ago was unremarkable.

Physical examination revealed mild tenderness on the right side of the mandible; however, there was no palpable mass in the neck or lymphadenopathy. Laboratory evaluation revealed leukocytosis, mild normocytic anemia, and notably abnormal liver tests (Table 1).

**Table 1:** Blood tests WBC: white blood cells; AST: aspartate aminotransaminase; ALT: alanine aminotransferase

Tests	Results	Units	Normal range
WBCs	11,400	/µL	4000-10900
Hemoglobin	12	g/dL	12-16
AST	153	IU/L	<41
ALT	109	IU/L	<54
Alkaline phosphatase	317	IU/L	38-126
Total bilirubin	1.8	mg/dL	0.2-1.2

**Figure 1:**
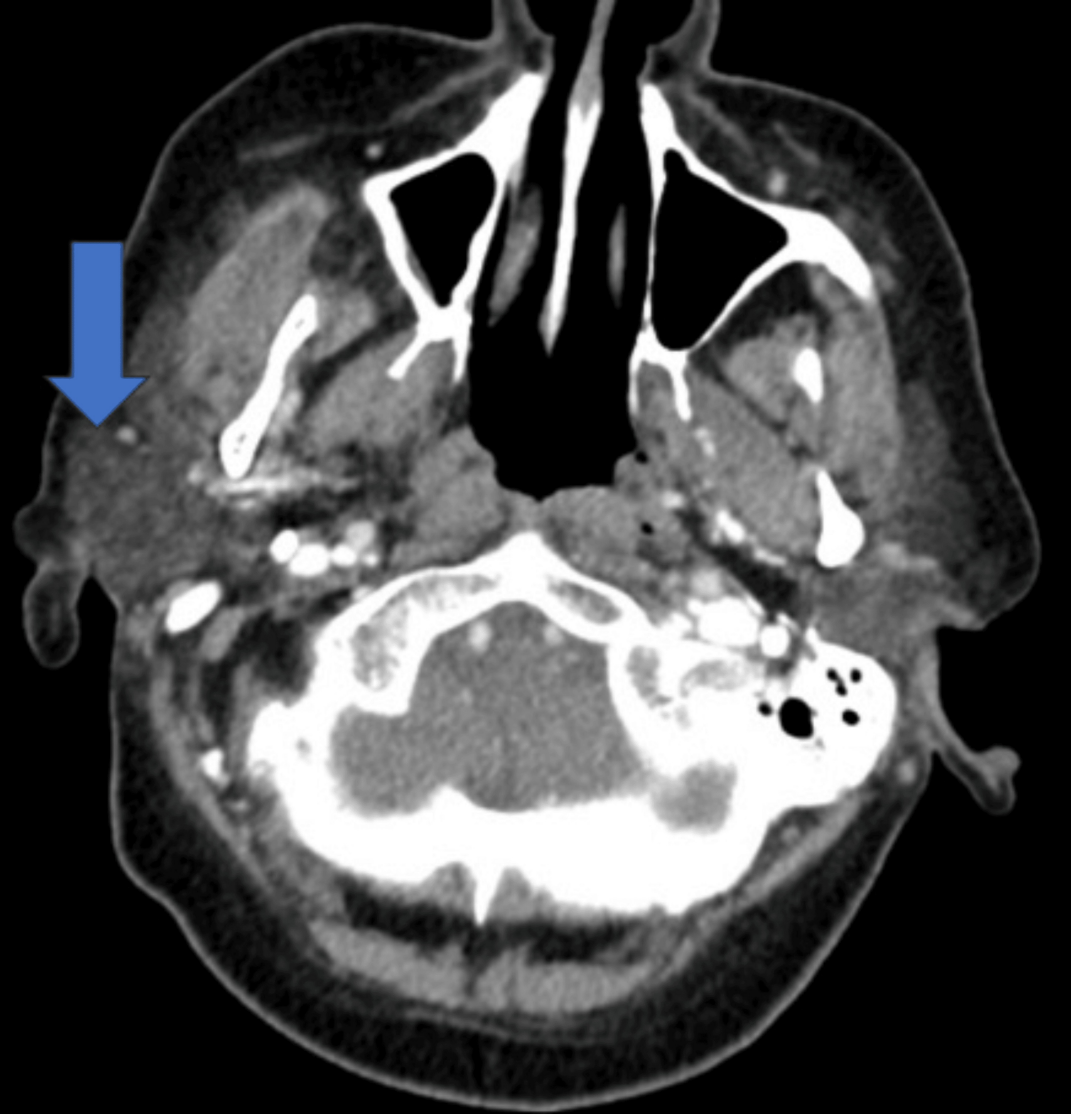
A helical CT scan of the neck reveals a large soft tissue mass accompanied by bony destruction of the right mandible (blue arrow)

A computed tomography (CT) scan of the neck was performed, revealing a large soft tissue mass with associated bony destruction of the right mandible (Figure 1).

**Figure 2:**
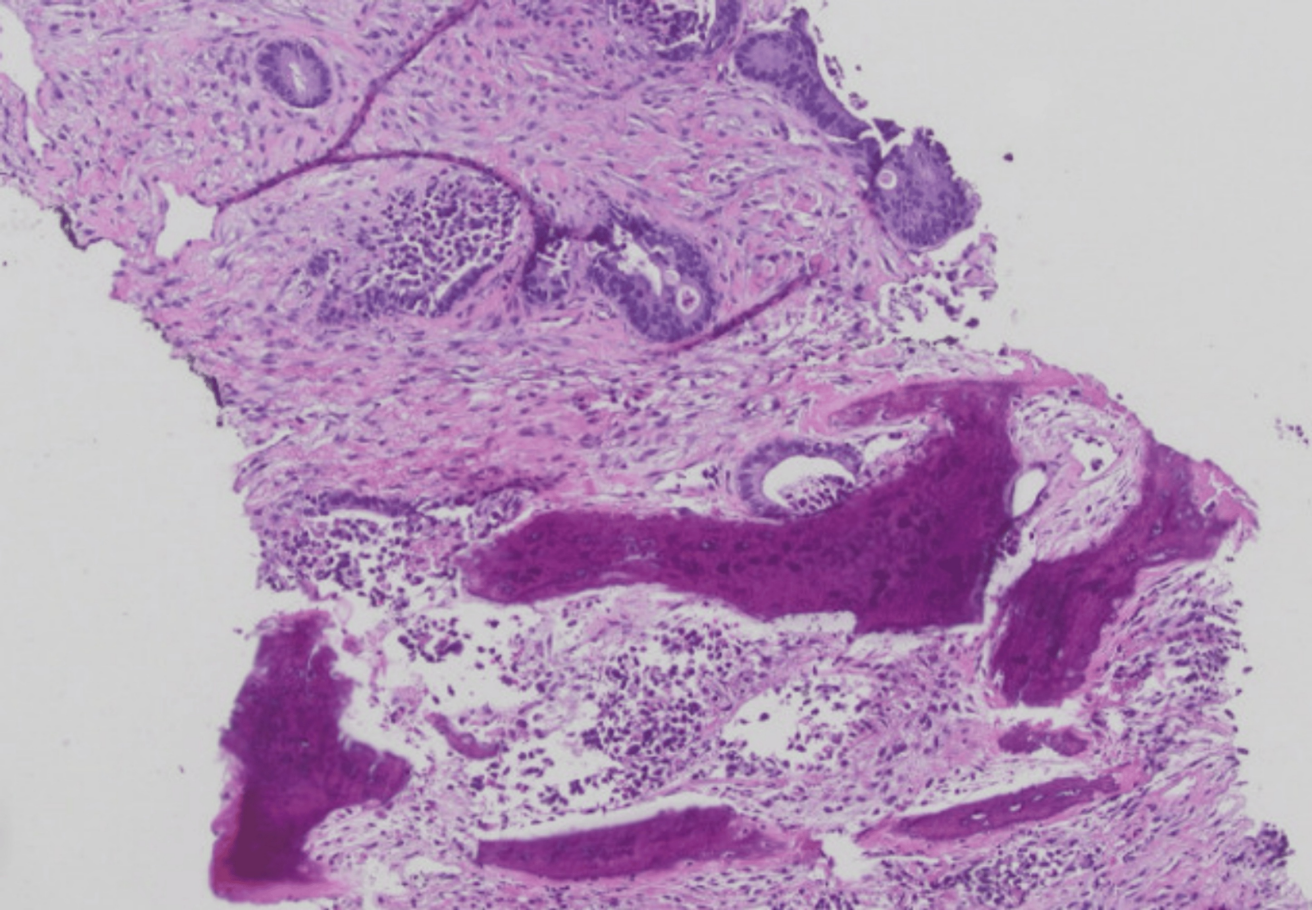
Jaw biopsy with H&E showing malignant cells H&E: hematoxylin and eosin

An ultrasound-guided biopsy of the lesion was performed. Histopathological analysis demonstrated malignant cells positive for cytokeratin AE1/3, CDX2, and SATB2, an immunoprofile consistent with metastatic adenocarcinoma of colorectal origin (Figure 2).

**Figure 3:**
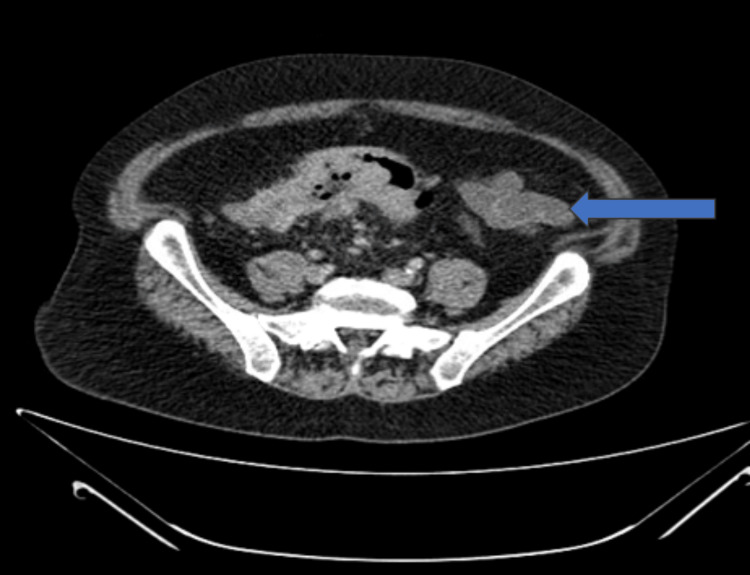
CT scan of the abdomen demonstrates thickening and surrounding fat stranding of the sigmoid colon (blue arrow)

Subsequently, a CT scan of the abdomen was performed due to long long-standing history of alteration in bowel habits to identify the primary malignancy, revealing segmental thickening and pericolonic stranding of the sigmoid colon, along with multiple low-attenuation lesions in the liver, suggestive of metastatic disease (Figure 3).

**Figure 4:**
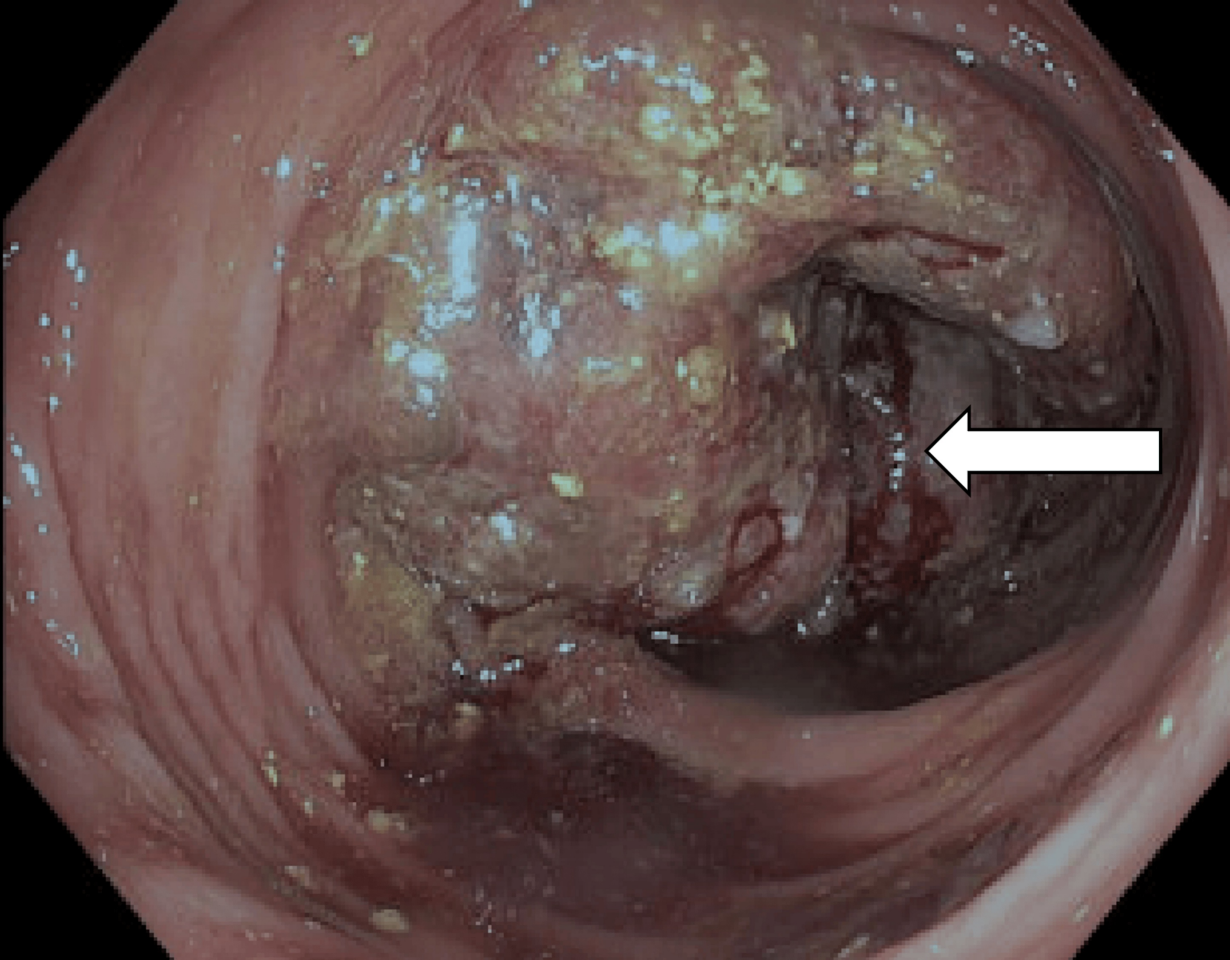
Colonoscopy reveals a 10 cm fungating mass in the sigmoid colon, partially obstructing the lumen (white arrow)

To localize the primary malignancy, a subsequent colonoscopy was performed, which identified a 10 cm fungating, partially obstructing mass in the sigmoid colon (Figure 4).

**Figure 5:**
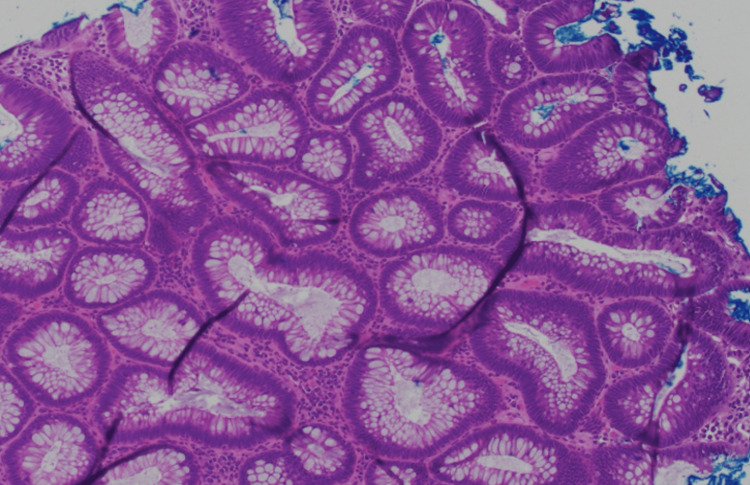
Sigmoid colon mass biopsy H&E showing invasive poorly differentiated adenocarcinoma H&E: hematoxylin and eosin

Histopathological analysis of the biopsy specimen confirmed a diagnosis of poorly differentiated invasive adenocarcinoma arising from a background of tubulovillous adenoma with high-grade dysplasia, with intact expression of mismatch repair proteins including MLH1, MSH2, MSH6, and PMS2 (Figure 5). Notably, carcinoembryonic antigen (CEA) levels were markedly elevated at 1666 ng/mL.

Given the extent of metastatic disease, surgical intervention was not considered viable at this stage. Subsequently, he was started on systemic chemotherapy with the folinic acid, 5-fluorouracil, and oxaliplatin (FOLFOX) regimen, in combination with bevacizumab. He was doing better during follow-up visits. Notably, CEA levels showed a significant reduction to 873 ng/mL, indicating an initial response to the treatment. He was doing better on initial follow-up office visits.

## Discussion

Approximately one-fourth of oral metastases serve as the initial sign of an undiagnosed primary malignancy located elsewhere in the body [8]. Oral metastatic tumors can occur in the jawbones or soft tissues; however, the jawbones are more frequently involved than the soft tissues [8]. The lungs are the most frequent primary origin of metastases to the oral soft tissues, while the breast is the most common source of metastases to the jawbones [5,6]. Isolated bone metastases from CRC are rare and typically occur alongside liver or lung metastases [8], as observed in our patient, who presented with both a jaw lesion and liver involvement. The mandible, particularly its posterior region, is more commonly affected due to its rich vascular supply. The proposed mechanism of spread is hematogenous dissemination, wherein circulating tumor cells seed the highly vascularized bone marrow of the jaw [9]. 

Patients typically present with jaw pain and swelling in the affected area, often mimicking odontogenic infections or other benign conditions. Diagnosis relies on imaging, biopsy, immunohistochemistry, and molecular profiling to determine the primary site. CT or magnetic resonance imaging (MRI) may show osteolytic lesions; however, in approximately 5% of cases, imaging may not reveal any abnormalities [9]. Technetium-99m bone scans or positron emission tomography-CT (PET-CT) can help detect bone metastases [10]. In our patient, immunohistochemistry confirmed colorectal origin through markers such as CDX2 and SATB2, which are known for their sensitivity and specificity in CRC [11].

To the best of our knowledge, fewer than 50 cases of metastatic colon cancer to the jaw, mandible, maxilla, tongue, or gingiva have been reported in the medical literature. Most patients were diagnosed in their 60s or 70s, with a male predominance. In 10 (22%) of these cases, the metastasis was diagnosed before the primary tumor was discovered-our patient represents the eleventh such case [12].

Systemic therapy remains the mainstay of treatment for metastatic colorectal cancer. First-line regimens typically include combination chemotherapy (e.g., FOLFOX, folinic acid, 5-fluorouracil, and irinotecan (FOLFIRI)) with targeted agents such as anti-epidermal growth factor receptor (EGFR) monoclonal antibodies for RAS/RAF wild-type tumors or anti-vascular endothelial growth factor (VEGF) agents like bevacizumab across molecular subtypes. While these approaches can extend median survival to 20-30 months in selected patients, outcomes are significantly worse in those with bone and jaw involvement. Jaw metastases are associated with poor prognosis, often indicating widespread disease [13-15]. Local radiotherapy may offer symptomatic relief but does not improve survival [16,17]. Antiresorptive agents can reduce skeletal complications but carry a risk of osteonecrosis [18]. Overall, treatment in such cases is palliative, with median survival typically less than one year [19]. Multidisciplinary management is critical for optimizing symptom control and quality of life.

## Conclusions

In conclusion, oral metastatic lesions from adenocarcinoma are exceedingly rare. The mandible is the most common site of oral bony metastasis. It should be included in the differential diagnosis depending on the clinical presentation, even in the absence of a known primary tumor, since such lesions may be the first manifestation of an occult malignancy. A timely diagnosis is crucial, and management primarily involves palliative chemotherapy, which can improve quality of life.
